# The Effectiveness of Attention Bias Modification with and without Trans Cranial Direct Current Stimulation in Chronic Low Back Pain

**Published:** 2020-04

**Authors:** Yasaman Shiasy, Shima Shakiba, Farhad Taremian, Seyed Majid Akhavan Hejazi, Alireza Abasi

**Affiliations:** 1 Department of Clinical Psychology, University of Social Welfare and Rehabilitation Sciences, Tehran, Iran*.*; 2 Substance Abuse and Dependence Research Center, University of Social Welfare and Rehabilitation Sciences, Tehran, Iran.; 3 Physical Medicine & Rehabilitation Specialist, Rofeideh Hospital, University of Social Welfare and Rehabilitation Sciences, Tehran, Iran.

**Keywords:** *Attention Bias Modification*, *Chronic Low Back Pain*, *Randomized Control Trial*, *Transcranial Direct Current Stimulation*

## Abstract

**Objective:** The present study aimed to compare the effect of ABM (attention bias modification) with and without tDCS (transcranial direct current stimulation) on attention bias, pain intensity, and disability due to pain and pain-related psychological consequences, such as depression, anxiety, and stress.

**Method** **:** Using convenience sampling, 60 individuals who met the criteria for chronic low back pain (LBP) were selected and randomly assigned in to 2 experimental groups and 2 control and sham-tDCS groups. The experimental ABM group received 5 sessions of the dot-probe task, while the second experimental group received 5 sessions of dot-probe task combined with tDCS.

**Results: **The findings indicated that ABM and ABM+tDCS could reduce attention bias and pain-related psychological consequences significantly, compared to the control and sham groups. Also, attention bias and pain outcomes (depression, anxiety, disability due to pain and pain intensity) remained in ABM+tDCS group than in ABM group in a 1-month follow-up.

**Conclusion: **It was found that tDCS + ABM had no additional effects at the end of intervention, but led to more long-lasting effects in 1-month follow-up.

Randomized clinical trial registry number: IRCT20171107037306N1.

According to the guidelines, chronic pain is described as the pain of any etiology correlated with a chronic medical condition or increase in duration beyond the expected temporal border of tissue damage and healing, unfavorably affecting the function of the patient (1). In the studies on this topic, attention bias to pain-related information is also the subject of major investigation activity in pain (2-4). Attention bias, or preferably attending to information that is related to the content of the sentimental concerns of patients, has confirmed to be a vigorous phenomenon in several forms of psychopathology (5-7). During the recent years, a large body of research has focused on evaluating the role of attention biases in experiencing pain, and some systematic reviews (8, 9) and meta-analyses (10, 11) have been conducted in this regard. Haggman et al (12) showed a bias to sensory pain words in patients with chronic and acute pain, but not in a matched group of normal cases. Biases have also been found in some researches on patients with chronic headaches (13, 14).

Moreover, 2 more recent meta-analyses have proven that patients with chronic pain exhibit small to moderate attention biases to pain stimulus, at least toward sensory pain words (15, 16). These were taken as proof that patients with chronic pain demonstrate an attention bias to pain-related information.

Such results have led to a new intervention that is based on modifying attentional biases, termed as attention bias modiﬁcation (ABM). Using the dot-probe paradigm (17), ABM was shown to significantly increase cold pressure pain thresholds in healthy adults (18-20) and decrease pain intensity and frequency among adults with acute back pain.

Thus, based on the preliminary evidence (21), ABM may play a role in improving pain and pain-related outcomes, although the findings of different studies are contradictory, which necessitates conducting more randomized controlled trials (9).

Transcranial direct current stimulation (tDCS) (22) has been established as an encouraging tool in neuroscience studies for insight into the relationship between behavior and brain in healthy people and patients (23). It acts by altering the excitability of neurons (24, 25), especially by inhibiting and activating the cortical circuits in the case of nerve stimulation of cathodal and anodal, respectively. Also, tDCS has been reported to reduce pain in patients who present with ﬁbromyalgia (26), traumatic spinal cord injury (27), and cancer (28).

Various studies have shown that when applied to cognitive rehabilitation treatments, such as attentional bias modification, electrical stimulation increases the effectiveness of these interventions (29, 30).The application of such hybrid protocols is not limited to linguistic and motor functions and can encompass a range of cognitive functions such as attention.

To date, few studies have combined 2 interventional strategies: postsurgical and laboratory-induced pain (31). To the best of our knowledge, no study focused on the impact of tDCS, combined with a cognitive intervention, on the prefrontal cortex. The present study aimed to evaluate the effectiveness of ABM alone and in combination with tDCS on attention bias, pain severity, disability due to pain and depression, stress, and anxiety among patients with chronic low back pain (CLBP).

## Materials and Methods

***Design***

A randomized controlled trial with single-blind design, parallel-groups, and one-month follow-up was conducted to assess effect of ABM with and without tDCS on attention bias and pain-related psychological consequences. The present study is designed based on the principles set in the Declaration of Helsinki (32) and was accepted by the (USWR). Randomized control trail registration was done on the website http://www.irct.ir/. (Registration number: IRCT20171107037306N1).

**Figure 1:**
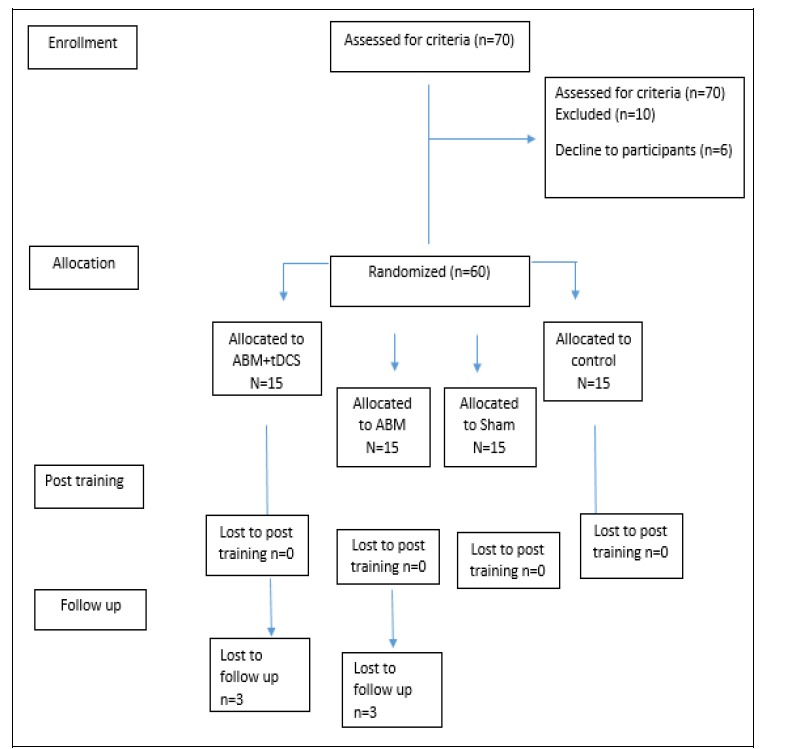
CONSORT (Consolidated Standards of Reporting Trials) Diagram of the Progress through the Phases of the Randomized Trial

Setting:

Among 70 patients with LBP who were selected through convenience sampling by a specialist in physical medicine in Rofeideh hospital, 60 patients met the inclusion and exclusion criteria and were randomly assigned into the groups. Participants were notified about the aim of the research and completed the consent form. The intervention was done for groups in Rofeideh rehabilitation hospital by a researcher blind to group status over 3 steps assessments, including pretest posttest, and 1-month follow-up. An online research randomizer program was used (www.randomizer.org). To ensure blindness, participants were randomly and blind assigned to one of 4 groups, including ABM-500 (n = 15), ABM+ tDCS (n = 15), –Sham- tDCS (n =15), and control group (n = 15). Figure 1 summarizes the trial CONSORT (consolidated standards of reporting trials) diagram.

***Inclusion Criteria***

Inclusion criteria were experiencing chronic LBP (lasting for more than 3 months) and age between 18 and 60 years. 

***Exclusion Criteria***

Having an epilepsy diagnosis or any chronic psychiatric pain disorders or receiving treatment for any psychiatric disorder, either currently or within the past 5 years were exclusion criteria. Also, those who were taking any psychiatric medication were excluded from the present study.

***Procedure***

After obtaining permission from the University Ethics Committee and recording the research at the clinical trial site (IRCT20171107037306N1), the researchers began a 5-step sampling process at Rafideh hospital: Patient selection, pretest, intervention, posttest, and 1-month follow-up. All procedures were performed individually for each participant. Patients who referred to physical medicine and rehabilitation were examined by a physical medicine specialist and referred to the examiner if they met the inclusion criteria. All participants were matched according to demographic variables (occupation, age, marital status, duration of LBP, and type of drug used) and were divided into 4 groups. In the first evaluation session, the questionnaires of pain intensity, pain disability, attention bias, and depression-anxiety, and attention bias (using dot-probe) were administered.

Intervention sessions were then started for 24 hours and continued for 5 consecutive sessions. At the last session, the same questionnaires were re-evaluated. A total of 70 individuals entered the study, of whom 60 remained in the study at all stages but 10 withdrew. All stages of evaluation and intervention were performed in Rafideh hospital. Moreover, to prevent any potential hazard to people receiving electrical stimulation (tDCS), a researcher-designed questionnaire and risk items were extracted using review articles. Brochure treatment was also provided to inform patients about this treatment and information was provided on this treatment. 

***Attention Bias Test (Dot-Probe Task)***

To assess attention bias, we used a modified form of the probe classification version of the dot-probe paradigm (MacLeod, Mathews, and Tata; 1986) (33), which was administered using Sina software. The stimuli used in the dot-probe test were pain-related images to establish each bias to attend to the location of pain-related pictures, neutral pictures. The reason for the use of images instead of words was that the patients with chronic pain may think more about pain or talk about it with different people, including specialists, friends, and family; thus, pain-related words were attracted more attention due to their greater use. Therefore, the observed effect of attention bias in studies using words as stimuli may indicate more familiarity and more frequent usage by patients. The other limitation of utilization of words is that many words contain several meanings, including a meaning which is not directly related to pain (34, 35). To prepare dot-probe images, 100 pain-related images were initially prepared and evaluated by 10 clinical psychologists and 10 chronic pain patients using self-assessment Manikin in dimensions of arousal and pleasure (36). Finally, the common images that received the highest scores on the arousal scale and the lowest scores on the pleasure scales were selected. In this scale, the response time of a person to neutral stimuli and pain-related stimuli were calculated separately. The attention bias score was equal to the difference between these values. If the reaction time of the person to the pain-related stimuli were less than the response time of the individual to the neutral stimulus, the person suffered from attention bias. 

***Brief Pain Inventory (BPI; Cleeland & Ryan, 1991)***

The BPI is a usual used instrument for measuring pain intensity and interference. Pain intensity was assessed via 4 items asking patients to rate their worst pain, least pain, and average pain over the past week, along with their current pain, on an 11-point rating scale, with (0 indicating no pain and 10 the worst pain (37). The average of these 4 items is calculated to form the Pain Intensity Scale (PIS; recommended by the IMMPACT consensus group). Pain interference was assessed via 7 items asking the degree to which pain has interfered with general activity, mood, walking ability, normal work, relations with other people, sleep, and enjoyment of life over the past week, which was evaluated on an 11-point rating scale (0 = no interference, 10 = complete interference). The average of these 7 items is calculated to form the PSI. Also, the BPI includes a single item assessing the extent to which treatments and medications have caused to relieve pain over the past week and a body map. Thus, patients can graphically indicate the location of their pain. The psychometric properties of the BPI are well-supported by involving the internal consistency of pain intensity and interference items (r = .85 and .88, respectively) (37, 38). Cronbach’s alpha for the sample was .92 for the total score at baseline.

***Roland Morris Disability Questionnaire (RDQ; Roland & Morris 1983)***

RDQ is a self-reported test, which was first published in 1983. The questionnaire provides a tool for measuring the level of disability experienced by a person suffering from LBP. Since then, it has become one of the most widely used outcome measures for LBP. The original 24-item measure was shortened to create 18-item and 23-item versions and has been cross-culturally adapted or translated for use in other countries. Both internal consistency (α = 0.84 to 0.96) and test-retest reliability (r = 0.83 to 0.91) of the RDQ seem appropriate for data collection. Also, it has a moderate to large correlation with other self-reported disability questionnaires, such as the Quebec Back Pain Disability Scale (Quebec Scale) (r = 0.6) and the Oswestry Disability Index (r = 0.5) (39). Cronbach’s alpha for the sample was 0.75 for the total score at baseline.

***Depression Anxiety and Stress Scales (DASS; Lovibond & Lovibond, 1995)***

The DASS is a 21-item self-report measure with 3 subscales employed for assessing depression, anxiety, and stress. Each subscale includes 7 items, which are rated on a 4 point Likert scale (0 = not at all to 4 = very much) (40). The validity of the questionnaire was already confirmed in the previous study by having a good internal consistency and convergent and discriminant validity (41). In this study, internal consistency was for the total scale (α = 0.94) and all 3 subscales (r = 0.88, for depression, r = 0.83 for anxiety, r = 0.90 for stress).

***Interventions***

***tDCS***

Five 20-minute tDCS (made by Medinateb company, Model: Tes-2ch, Serial Number: MD2-8037) sessions were conducted using 2.0 mA current. Electrodes were 4_4 cm saline-soaked sponge electrodes. The electrode size was selected to increase the focal administration of tDCS current or minimization of the spread of applied current to brain areas outside of the targeted area. Anode electrode was applied to the left prefrontal cortex (M). M1 was usually defined as the location of the C3/C4 electrode in the International 10–20 system for EEG Electrode placement. 

Regarding another electrode (cathode), it was placed on the contralateral side supraorbital and both were held in place by elastic bandages. Electricity was slowly increased to 2 mA at the beginning of the stimulation (30 seconds fade in) and slowly decreased at the end of the stimulation (30 seconds fade out) (42)

Regarding sham stimulation, the device switched off automatically after a 30-second period of stimulation, which mimicked the tingling or mild burning sensation commonly perceived by the participants. This brief period of stimulation does not lead to any neurophysiological changes (43).

***ABM***

First, the 50 pairs of pictures were randomly presented 8 times in each of the 4 possible combinations for 500 milli second (left arrow top/target top; right arrow top/target top; left arrow bottom/target bottom; right arrow bottom/target bottom). Then, they were instructed to focus on the center and all parts of the screen and say as quickly and accurately as possible whether they see a left or right facing arrow on screen using the corresponding arrow keys on the keyboard (The arrow probe disappeared when it was keyed in or after 1-s). In the next procedure, the identity of the arrow probe was randomized for each trial. No indication was given to the participant that the ABM procedure might influence their pain experience.

***Statistical Analysis***

All analyses were conducted using SPSS 23.0 software. First, 1-way ANOVA and chi-square tests were used to determine whether there were any differences between the 4 randomized groups (ABM, Sham-tDCS, ABM+tDCS, and control) on demographic and outcome variables. The MANCOVA and repeated measure tests were used to investigate the effect of the intervention on attention bias score and pain-related psychological outcomes. The assumptions of MANCOVA and repeated measure tests, including the study of homogeneity of variance, were investigated and confirmed.

## Results

For descriptive statistics, please see Table 1. Based on the results of chi-square, no significant difference was observed between the groups at baseline in marital status (X2(3), N = 60, 57.60, p = 0.56) Job (X2(4) N = 60, 18.54, p = 0.12) and Drug X23, N = 60 = 31.06, P = 0.20). Based on 1-way ANOVA results, no significant differences were observed in terms of age (F (3) = 5.79, p = 0.12), and the duration of LBP (F (3) = 10.67, p = 0.78).

***Attention Bias***

Based on the results of the MANCOVA test, the effect of the group or intervention on attention bias was statistically significant (F (3) = 13/9, p < .05, ƞ2 = 0.47); therefore, the independent variable caused a difference among the groups. The results of the Bonferroni test demonstrated that the difference between the ABM and the combined groups was not meaningful. Further, no significant relationship observed between control and sham groups. The ABM group was significantly different from the control and sham groups.

Moreover, the combined group had a significant difference with both control and sham groups. In general, a significant difference between the ABM with and without tDCS and both control and sham groups showed reduction in attention bias. For a more comprehensive image see Table 2.

***Pain Intensity***

Based on the results, the groups showed a statistically significant association with the pain intensity (F (3) = 13/09 p < 0.05, Ƞ2 = 0.47); consequently, the independent variable made the difference between the groups. Subsequently, the Bonferroni test was applied to compare the differences between the groups and the results indicated no significant difference between the ABM and combined groups and a significant difference between the ABM and combined groups and the sham and control groups. Also, there was a significant difference between the control and sham groups. Generally, the ABM with and without tDCS had a significant impact on pain reduction, compared to the control and sham groups (For a more comprehensive image see Table 2).

***Pain-Related Disability***

The results of the MANCOVA test revealed a statistically significant effect of the group on the pain-related disability (F (3) = 9.36, p < 0.05, Ƞ2 = 0.39). Accordingly, the independent variable led to the difference between the groups. In the following, the Bonferroni test was used to compare the difference between the groups. There was no significant difference between the ABM and combined groups. Furthermore, a significant difference was observed between the ABM and combined groups and the sham and control groups. In general, the ABM with and without tDCS had a significant effect on the reduction of pain-related disability, compared to control and sham groups (For a more comprehensive image see Table 2).

***Depression, Anxiety, and Stress***

The results indicated an insignificant impact of the group on the subscale of depression (F (3) = 0/08, p > 0.05, Ƞ2 = 0/26), and a significant impact of the group on the subscale of anxiety and stress. The pretest score influenced the anxiety and stress subscales in the posttest, and some of the variances of the posttest scores was influenced by the pretest. Then, the effect of the pretest as covariance factor was eliminated to examine the impact of the group or intervention on the subscale of depression. Based on the results, the effect of the group on the subscale of anxiety was statistically significant (anxiety: F (3) = 6/64, P < 0.05, Ƞ2 = 0/31; stress: F (3) = 3/69, p < 0/05, Ƞ2 = 0/21). (For a more comprehensive image see Table 2).

**Figure2:**
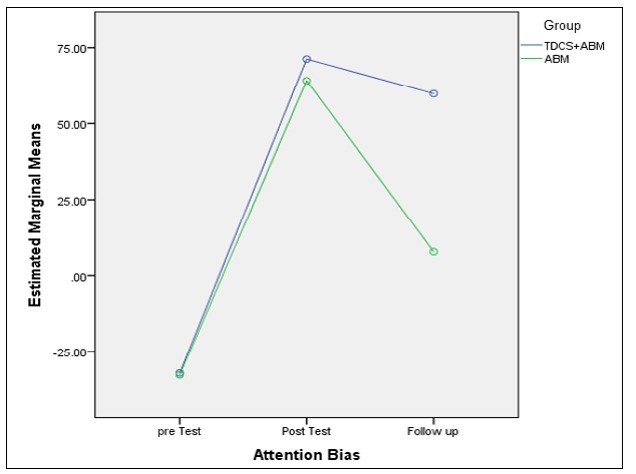
Comparison between ABM and ABM + tDCS Groups in the Follow-Up

**Comparing the ABM and ABM + tDCS Groups Regarding Maintaining the Treatment Achievement** There was a significant difference in attention bias in both groups in 3 stages of measurement (F (2) = 26.11, P < 0.05). The results of the groups comparison indicated no significant difference between the 2 groups in the attention bias variable (F (1) = 1.19, p > 0.05). Also, there was a significant difference in attention bias between pretest and posttest stages, while no significant difference was observed between the pretest and posttest stages. Thus, the results had no significant variation during 1 month. Figure 2 illustrates that the individuals’ attention bias score in the combined group was more stable in the follow-up stage.

**Figure 3:**
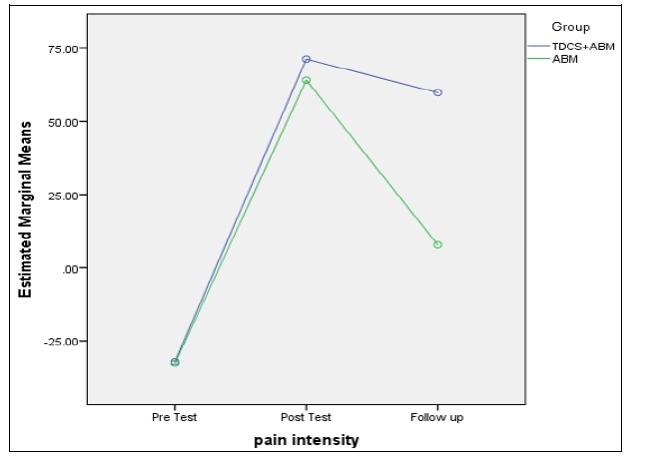
Comparison between ABM and ABM + tDCS Groups in the Follow-Up

Pain intensity was significantly different in both groups at all 3 measurement stages (F (2) = 36.53, p < 0.05). The results of the groups comparison demonstrated no significant difference between the 2 groups (F (1) = 2.99, p > 0.05). Figure 3 illustrates that the individual’s attention bias score in the combined group was more stable in the follow-up stage.

**Figure 4:**
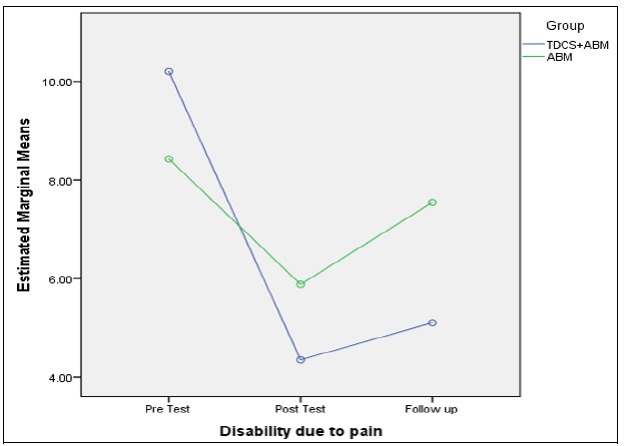
Comparison between ABM and ABM + tDCS Groups in the Follow-Up

Also, the score of the pain-related disability in the measurement stages was significantly different (F (2) = 14.82, p < 0.05). The results of the group comparison indicated no significant difference between the groups (F (1) = 0.33, p > 0.05). Furthermore, pain-related disability had a significant difference in the pretest and posttest stages, while no significant difference was reported between the posttest and follow-up stages. Thus, the changes maintained during 1 month. Figure 4 demonstrates the more stable variations of the combined group in the follow-up stage.

**Figure 5:**
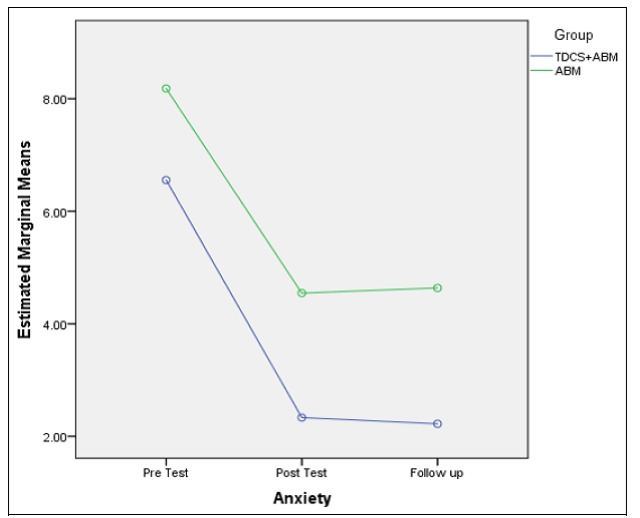
Comparison between ABM and ABM + tDCS Groups in the Follow-Up

**Figure 6:**
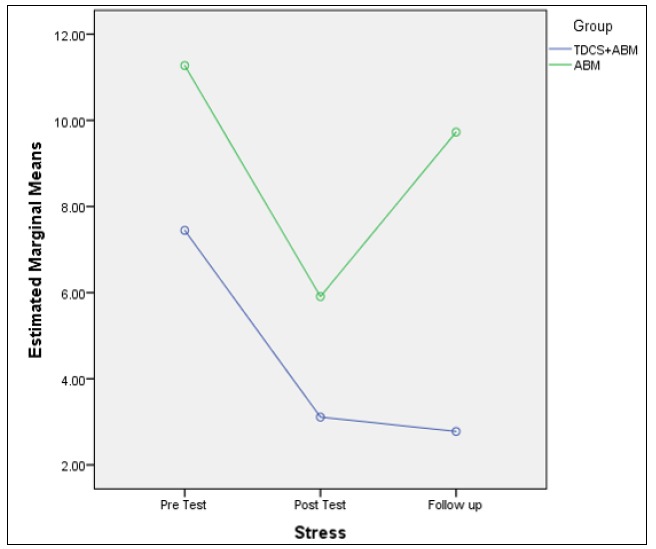
Comparison between ABM and ABM + tDCS Groups in the Follow-Up

The score of the anxiety and stress in the measurement stages were significantly different (anxiety: F (2), 21/77, p < 0.05; stress: F (1), 6/13, p < 0/05). The results of the group comparison indicated no significant difference between the group (anxiety: F a (1), 2/60, p > 0/05; stress: F (1), 3.66, p > 0/05). Furthermore, anxiety and stress had a significant difference in the pretest and posttest stages, while no significant difference was reported between the posttest and follow-up stages. Thus, the changes maintained during 1 month. Figures 5 and 6 demonstrate the more stable variations of the combined group in the follow-up stage. Table 3 shows the results in detail.

***Report Reliable Change Index (RCI)***

RCI was used to examine clinical significance. Cutoff point was obtained by calculating the RCI to determine the therapeutic change status of each individual in the treatment group in both directions of positive therapeutic outcomes and possible negative symptoms and outcomes of treatment. The calculation of the RCI for each variable was based on the standard deviation of the variable score before and after the treatment as well as the internal consistency coefficient of the variable.

Regarding the pain intensity variable, 20 participants had a meaningful clinical variation, half of the participants belonged to the combined group, and the other 10 were the members of the attention bias group. The percentage of people with a meaningful variation in both groups was 0.66%. In the pain-related disability variable, 23 participants had a meaningful clinical change, of whom 13 and 10 belonged to the combined and attention bias modification groups, respectively. The percentage of changes in the groups (combined, attention bias) was 0.86 and 0.66, respectively. In the anxiety variable, 22 participants achieved significant clinical changes, 8 of them were in the attention bias group, while the rest belonged to the combined group. The percentage of the clinical changes of individuals was 0.93 and 0.33 in the combined and attention bias group, respectively.

Finally, regarding the stress variable, 22 participants achieved significant clinical changes; 9 of them were in the attention bias group and 13 in the combined group. The percentage of individuals’ changes was 0.86 in the combined group and 0.60 in the attention bias group.

**Table 1:** Descriptive Statistical Indicators (Means and SD) for Baseline Characteristics and Pain-Related Psychological Consequences

	**ABM**	**ABM+tDCS**	**Sham**	**Control**
	**M (±SD)**	**M (±SD)**	**M (±SD)**	**M (±SD)**
**Age**	35.6 (12.44)	35.6 (8.77)	30.50 (10.66)	28.92 (11.92)
**Attention Bias**	-27.73 (18.82)	-29.20 (19.79)	-35.93 (24.71)	-33.60 (21.09)
**Pain Severity**	40.4 (16.41)	56.13 (17.28)	42.86 (18.58)	24.66 (16.88)
**Pain-related disability**	10.24 (4.81)	12.60 (4.93)	9.46 (3.44)	9.20 (3.14)
**DASS-depression**	8.06 (1.18)	7.21 (0.93)	8.61 (1)	6.08 (0.68)
**DASS-Anxiety**	7.93 (1.18)	6.14 (0.73)	5.69 (1.02)	4.91 (0.78)
**DASS-Stress**	10.40 (1.26)	7 (0.95)	5 (0.69)	6.83 (0.90)

**Table 2:** MANCOVA Test Results for Outcome Measures (Attention Bias, Pain-Related Disability, Pain Intensity and DASS)

**Measure**	**Effect**	**Df**	**F**	***p***	**ɳ** ^2^
**Primary Outcome**					
**Attention Bias**	Covariance	1	2/65	0/11	0/05
	Group	3	8/49*	0/001	0/37
	Error	43			
**Secondary Outcomes**					
**Pain Intensity**	Covariance	1	5/35*	0/02	0/11
	Group	3	13/09*	0/001	0/47
	Error	43			
**Pain-related Disability**	Covariance	1	23/20*	0/001	0/03
	Group	3	9/36*	0/001	0/39
	Error	41			
**DASS-Depression**	Covariance	1	15/79*	0/01	0/26
	Group	3	4/52	0/08	0/26
	Error	43			
**DASS-Anxiety**	Covariance	1	9/66*	0/003	0/18
	Group	3	6/64*	0/001	0/31
	Error	43			
**DASS-Stress**	Covariance	1	2/12	0/15	0/04
	Group	3	3/69*	0/01	0/21
	Error	43			

NOTE: DASS: Depression-Anxiety Stress Scale

* p < 0.05

**Table 3:** Repeated Measure Test Results for Outcome Measures (Attention Bias, Pain-Related Disability, Pain Intensity and DASS) in the Follow-Up

**Measure**	**Effect**	**df**	**F**	***p***	**ɳ** ^2^
**Primary Outcome**					
**Attention Bias ** **Within subject**	Time Error	2 19	26/11*	0/001	0/57
**Between subject**	Group	1	8/49	0/001	0/37
	Error	19			
**Secondary Outcomes**					
**Pain Intensity** **Within Subject**	Time Error	2 38	36/5*	0/001	0/65
**Between Subject**	Group	1	0/94	0/005	0/05
	Error	19			
**Pain-related disability ** **Within Subject**	Time Error	2 38	14/8*	0/001	0/43
**Between Subject**	Group	1	0/33	0/57	0/01
	Error	19			
**DASS-Anxiety** **Within Subject**	Time Error	2 36	21/77*	0/001	0/54
**Between Subject**	Group	1	2/60	0/12	0/12
	Error	18			
**DASS-Stress** **Within Subject**	Time Error	1 36	6/13*	0/01	0/25
**Between Subject**	Group	1	3/66	0/07	0/16
	Error	18			

NOTE: DASS: Depression-Anxiety Stress Scale

* P < 0.05

*: After further investigation, 5 individuals were excluded because they failed to meet the inclusion criteria and 5 were also excluded due to withdrawal from the study.

## Discussion

This study set out to compare the effect of the ABM training with and without tDCS on the attention bias and pain-related psychological outcomes and to compare the maintenance of the treatment outcomes in follow-up. The primary results illustrated a significant difference in both experimental groups, compared to the control and sham groups.

The ABM training reduced the participants’ attention bias scores with LBP. Based on the results, a significant difference was observed between the attentional bias scores in the pretest and posttest in the ABM training group. Also, the ABM training had a significant effect on attention bias reduction. Expectedly, the results indicated that the ABM training could significantly reduce selective attention. The results were consistent with the findings of some studies (21, 44-47). In the same manner, the theory of the selective attention explains that selective attention is considered the initiator and continuator of anxiety, and even in the absence of a real threat, it is associated with physiological excitation (48). Continuous attention to threats-dependent stimuli affects the process of target-oriented behavior. The scope of the cognitive process about the attention allocation includes a vast network of automated and voluntary processes influenced by situational factors and individual differences (49). The ABM program in each session is designed to divert participant’s attention from the painful stimulus to the neutral stimulus successively, and therefore, the selective attention changes. In this regard, Lautenbacher (50) and Bar-Haim (51) argued that the effectiveness of the ABM-based treatments is due to the improvement of general attention control caused by these assignments. Heathcote et al (52) indicated that the effect of ABM on pain-related disability is due to the changes in attention controlling in which people change their attention from painful stimuli toward neutral stimuli. The results of frequent studies indicated that the patients with chronic pain tend to the threatening cognitive bias based on hypervigilance theory and selective choice and the manipulation of these processing biases leads to the effective results in the pain intensity (45). The interpretive style is modified by assignments made to evaluate the cognitive bias of ambiguous events. The cognitive bias modification method adjusts the biases through the educational conditions designed to manipulate the psychological harm-related processes (53). 

Martin et al (54) argued that the stimuli representing a natural hazard or a threat attract the initial attention to oneself, which allow processing or further details. However, not paying attention to threats or negative stimuli reflects the effort to regulate the mood, and individuals minimize the unpleasant feelings of these stimuli through cognitive avoidance. However, both hypervigilance and avoidance modes may be related to the pain and pain-related behavior in a different way. The supporting evidence derived from the recent studies indicated that the involvement of attention with pain-related stimuli is associated with higher levels of chronic pain (55). However, the avoidance of attention anticipates the postoperative pain-related outcomes (50). Thus, attention bias dimensions, vigilance, and avoidance are differently associated with the growth and continuation of pain. However, a flexible vigilance-avoidance pattern may be adaptive and negatively influence the pain in the exaggerated and apparent manner, because pain-related stimuli are detected very quickly and a bit late, are avoided and prevent adaptive coping. 

Taylor et al (56) examined brain activity of chronic pain patients during a dot-probe test using fMRI. Based on the results, ABM and attention retraining regulate the activity of areas associated with pain perception, and attention plays an important role in the process of pain. The pain experience is influenced by the way people pay attention to the pain-related stimuli. Therefore, attention-retraining methods play an important role in the perception of pain.

The primary motor cortex is the site of passage of the corticothalamic descending pathway, which is very effective in pain processing. Polania et al found that after anodic stimulation, motor cortex and thalamus function increased, which led to a decrease in pain intensity (57). There is some evidence that changes in cortical excitability induced by anodic cortex stimulation can be effective in relieving acute and chronic pain (58). Electrical stimulation can also cause changes in the concentration of glutamate and gamma amino butyric acid in the stimulated areas.

The results demonstrated no significant difference between the 2 treatments in the posttest stage, which are not in line with the results of the previous findings (30, 59-62), which indicated that the simultaneous combination of the cognitive rehabilitation protocols and electrical stimulation has a more significant effect, compared to the implementation of the protocol alone. This study was different from the previous studies in some areas. For example, they focused on people with stroke or head injury. However, the present study was the first research conducted on people with chronic back pain. The results of the follow-up process indicated that the changes in the score of participants were stable in both groups during a month although the severity of the changes was lower in the combined group. As electric stimulation provides the necessary background for various cognitive rehabilitation protocols by increasing the excitability rate of the cortex in the matrix-related networks, the findings can be related to the existence of a long-term reinforcement mechanism in the brain, resulting in a brief period of strong synaptic activity, which can lead to the continuous strengthening of synaptic transmission. This model is considered as the most accepted pattern for the infrastructural mechanism of learning and memory in the brain.

## Limitation

The current investigation was limited by using the dot-probe paradigm to measure and modify attention bias. Hence, it is suggested to use separate tools to measure attentional bias to better investigate the role of attentional bias and its impact on pain-related outcomes. In addition, a longitudinal study was not possible due to the lack of time, and only a 1-month follow-up was considered.

Thus, it is suggested to replicate this study with more precise tools, such as the use of QEEG and eye movement tracking-based methods, to evaluate the mechanisms of change. Considering that this was the first study done in patients with chronic LBP, it is recommended to repeat the same study in several samples of patients with chronic pain.

## Conclusion

It seems that the application of tDCS with cognitive rehabilitation using computer tasks through a long-term reinforcement mechanism improves the effectiveness of rehabilitation protocols (63). Although tDCS + ABM has no additional effects just at the end of intervention, it can lead to more long-lasting effects in 1- month follow-up.
